# Schooling Relates to Mental Health Problems in Adolescents with Cochlear Implants—Mediation by Hearing and Family Variables

**DOI:** 10.3389/fpsyg.2015.01889

**Published:** 2015-12-18

**Authors:** Maria Huber, Belinda Pletzer, Alexandros Giourgas, Andreas Nickisch, Silke Kunze, Angelika Illg

**Affiliations:** ^1^Department of Otorhinolaryngology, Head and Neck Surgery, Paracelsus Medical University SalzburgSalzburg, Austria; ^2^Department of Psychology and Center for Neurocognitive Research, University of SalzburgSalzburg, Austria; ^3^Department of Otorhinolaryngology, Hannover Medical SchoolHannover, Germany; ^4^Department of Hearing-Language-Cochlear Implants, kbo-Kinderzentrum MünchenMunich, Germany

**Keywords:** adolescents with cochlear implants, mental health problems, multicenter study, schooling, speech understanding in noise, SDQ, hearing loss

## Abstract

Aim of this multicenter study was to investigate whether schooling relates to mental health problems of adolescents with cochlear implants (CI) and how this relationship is mediated by hearing and family variables. One hundred and forty secondary school students with CI (mean age = 14.7 years, *SD* = 1.5), their hearing parents and teachers completed the Strengths and Difficulties Questionnaire (SDQ). Additional audiological tests (speech comprehension tests in quiet and noise) were performed. Students of special schools for hearing impaired persons (SSHIs) showed significantly more conduct problems (*p* < 0.05) and a significantly higher total difficulty score (TDS) (*p* < 0.05) compared to students of mainstream schools. Mental health problems did not differ between SSHI students with sign language education and SSHI students with oral education. Late implanted students and those with indication for additional handicaps were equally distributed among mainstream schools and SSHIs. However, students in SSHIs were more restricted to understand speech in noise, had a lower social background and were more likely to come from single-parent families. These factors were found to be partial mediators of the differences in mental health problems between the two school types. However, no variable could explain comprehensively, why students of SSHIs have more mental health problems than mainstream pupils.

## Introduction

Cochlear implants (CI) open the door for hearing impaired children to mainstream education (Waltzmann et al., 2002; Huber et al., 2008). If there are no further developmental risks or handicaps, the language development of very young implanted children with CI is very similar to that of their normal hearing peers (Spencer et al., 2004; Beadle et al., 2005; Uziel et al., 2007).

The input from the CI is the requirement for phonological awareness and phonological processing which is needed to decode words (Spencer and Tomblin, 2009) and is therefore a strong requirement for the development of oral language. Early listening and speaking skills predict in the long term reading skills of children with CI (Spencer and Oleson, 2008; DesJardin et al., 2009).

On the basis of cochlear implantation scores on academic tests are achieved that are within 1 SD of their hearing peers (Spencer et al., 2003, 2004).

Nevertheless, in European countries many hearing impaired children and adolescents with CI attend special schools for hearing impaired persons (SSHIs)^1^ (Meyer et al., 2013). In Germany still more than 50% of the hearing impaired children and adolescents with CI are in SSHIs. Only a limited number of German SSHIs (Illg et al., 2013) offer the same graduation as an academic high school, yet working with special educational plans.

Several studies indicate that mental health problems^2^ of hearing impaired children and adolescents are increased if they attend SSHIs compared to mainstream schools (van Eldik, 2005; Mejstad et al., 2009; Theunissen et al., 2014a). It concerns adolescents with sign language (Sweden: Mejstad et al., 2009; the Netherlands: van Eldik, 2005) as well as children and adolescents with hearing aids, the majority with oral language as mode of communication (Dutch, Theunissen et al., 2014a).

Also in a study including both hearing impaired children with and without CI, self esteem, which predicts mental health problems in normal hearing children (Ranøyen et al., 2015), was found to be significantly lower for pupils of SSHIs compared to mainstream pupils (Keilmann et al., 2007; Theunissen et al., 2014b). Regrettably the authors did not provide any information about the specific outcomes of the CI group. Therefore, little information is available so far on the relationship between schooling and mental health problems in adolescents with CI. As demonstrated in a recent study (Huber et al., 2015) mental health problems of adolescents with CI in general, particularly concern the interaction with peers. Apart from that the extent of emotional, behavioral and social problems of CI children, is comparable to that of normal hearing peers, if there are no additional handicaps.

In a study on the general prevalence of mental health problems among adolescents with CI in Austria, Huber and Kipman (2011) found that more emotional, behavioral and social problems were associated with SSHIs compared to mainstream schools.

Furthermore, no information is available on the reasons, why pupils of SSHIs may have more mental health problems than hearing impaired pupils of mainstream schools.

Therefore, the present study aims on the one hand to investigate the relationship between schooling and mental health problems in a large sample of hearing impaired adolescents with CI acquired in a German multi-center study. On the other hand, the study seeks to explore possible explanatory variables for differences in mental health problems between school types.

The potential reasons for these differences are manifold and may concern the following domains: (i) hearing variables, (ii) school variables, (iii) family variables.

First, problems in understanding and speaking may contribute to mental health problems. Studies about young hearing impaired persons without CI indicate that mental health problems are promoted by an unsatisfactory progress in speech- and language development (Barker et al., 2009; Stevenson et al., 2010) or by communication problems (Hogan et al., 2011). In the above-mentioned studies on self esteem, children and adolescents with poor auditory and oral speech outcomes were overrepresented in SSHIs (Keilmann et al., 2007; Theunissen et al., 2014b).

These differences in auditory and speech skills between school types may in part be due to selection processes. For example in Germany, before entering mainstream schools, the auditory and speech skills of hearing impaired pupils are estimated by teachers from SSHIs. Depending on the results, an educational plan is developed, which may be regular (for children without special needs) or special (for children with special needs). A child with a regular educational plan has the capability to attend a regular class.

Several variables have to be taken into account when assessing the auditory and speech skills of hearing impaired adolescents with CI as a mediator of mental health problems. In a school environment the ability to hear and to understand in noisy environments is surely of particular importance. There is an indication that problems to hear and to understand speech in noisy environments are associated with more mental health problems in young CI users (Huber et al., 2015).

Furthermore, one of the strongest predictors of hearing and speech skills during the first years after cochlear implantation is the age at which children receive their cochlear implant (Nikolopoulos et al., 1999; Sharma et al., 2002; Lesinski-Schiedat et al., 2004; Connor et al., 2006; Svirsky et al., 2007). Additionally it has to be taken into account that some children, e.g., those with Mondini Dysplasia, have congenital malformations of the inner ear, which complicates the cochlear implantation (Aschendorff et al., 2009). Language- and speech outcomes are variable in young CI users with such complications (Aschendorff et al., 2009; Black et al., 2014).

Second, differences in the school environment itself may contribute to more mental health problems. Among the most obvious differences between SSHIs and mainstream school are sign language education and boarding schooling. While in regular mainstream schools students are required to use oral language, sign language is an integral part of SSHI educational plans in most countries (Langereis and Vermeulen, 2015). German SSHIs offer classes with sign language, oral language, or total communication^3^. In the above-mentioned studies on mental health problems in hearing impaired adolescents at SSHIs, most of these adolescents did not grow up with oral language, but with “total communication” or “two-way” communication (Dutch: van Eldik, 2005; Sweden: Mejstad et al., 2009). Furthermore, SSHIs in Germany are very often residential schools. Normal hearing children who live in residential homes are more vulnerable for mental health problems than children who live at home (Bradley and Vandell, 2007).

Third, we suppose that children with additional needs mostly attend SSHI. This may concern multi- handicapped children. Hearing impaired children have an increased risk by about one third for additional handicaps, compared to normal hearing children (American Academy of Pediatrics, Joint Committee on Infant Hearing, 2007; Gallaudet Research Institute, 2011). Studies on hearing impaired children (van Eldik, 2005; van Gent et al., 2007) as well as normal hearing children (Carvill, 2001; Barkauskiene and Bieliauskaite, 2002; Dekker et al., 2002; Leask et al., 2002; Glazebrook et al., 2003; Hemmings et al., 2006; Kaptein et al., 2008; Backenson et al., 2015) indicate that disabling health conditions increase the risk for more mental health problems and disorders.

Furthermore, additional needs of children and adolescents in SSHIs may also be associated with a disadvantaged social background. In our clinical experience, hearing impaired children from disadvantaged social backgrounds are enrolled more often in special institutions, whereas children with a middle class background tend to grow up in mainstream institutions. Mental health problems are frequently associated with a low socioeconomic status in normal hearing children (Aebi et al., 2014), hearing impaired children (Theunissen et al., 2014a), as well as children with CI (Huber et al., 2015).

The first aim of the present study was to investigate whether schooling relates to mental health problems of adolescents with CI. The second aim was to determine possible differences in the variables listed above between SSHIs and mainstream schools and to investigate, whether these differences affect the relationship between school type and mental health problems.

Our hypotheses are the following:

- Adolescent CI users who attend secondary SSHIs show more mental health problems than adolescent CI users who attend regular and integrative classes of secondary mainstream schools.
- Hearing variables: Participants with poor auditory and speech perception in quiet as well as with restricted auditory performance in noise, late implanted participants (age at implantation of the 1st CI equal or higher than 5 years) and participants with indication for additional handicaps are more likely to attend SSHI.
- School variables: Pupils of SSHIs are more likely to prefer sign language as their mode of communication, are more skilled in sign language and are more often educated primarily in sign language than pupils of mainstream schools. Furthermore, pupils of SSHIs attend more often residential schools than pupils of mainstream schools.
- Family variables: Pupils of SSHIs have a lower socioeconomic status (educational level and skill level of the parents) compared to pupils of mainstream schools. They live more often in a single-parent family.
- The variables listed in (b–d) explain at least in part the differences in mental health problems between mainstream schools and SSHIs.

The results of the study may be important for parents of hearing impaired children with CI in order to ensure the most optimal school for their child. Additionally, the results may be important for all teachers of students with CI to guide them and their parents. Furthermore, they may be important for the whole school system to set appropriate proposals for children with CI.

## Methods

This study is part of a more comprehensive Austrian and German project about mental health problems of adolescents with CI and was conducted as a multi-center study. The participating centers of the present study were: the Cochlear Implant Center Freiburg at the University of Freiburg, the Department of Otorhinolaryngology Hannover at the Hannover Medical School, the University Medical Center at the University of Mainz and the kbo-Kinderzentrum München (Socialpediatric Center) in Munich.

### Participants

The total group comprised 140 adolescents with CI (68 boys, 72 girls) and their hearing parents and teachers, 30 from Freiburg, 43 from Hannover, 44 from Mainz and 23 from Munich (all Germany). Our response rate was 79% out of 178 possible cases^4^. All adolescents were between 12 and 17 years old (mean age = 14.72 years, *SD* = 1.51 years), were diagnosed with severe or profound hearing loss before the age of 24 months and had been using their first CI for at least 3 years. Demographic data of the total group can be found elsewhere (Huber et al., 2015).

At the time of investigation 83 out of 140 students with CI (59%) visited secondary SSHIs (47 girls, 36 boys, mean age = 14.78 ± 1.54 years). 38 out of 140 (27%) visited regular classes of secondary mainstream schools (18 girls, 20 boys; mean age = 14.63 ± 1.46 years) and 19 (14 %) went to integrative classes of secondary mainstream schools (7 girls, 12 boys, mean age = 14.71 ± 1.48 years). Age and gender did not differ significantly between school types. Among the 83 students at SSHIs mental health ratings were available from 77 students, 82 parents and 30 teachers. Among the 38 students who had visited a regular class at a secondary mainstream school, mental health ratings were available from 34 students, 38 parents and 15 teachers. For the 19 pupils in integrative classes of secondary mainstream schools 19 self ratings, 19 parent ratings and 10 teacher ratings were available. The demographic data and hearing variables of mainstream pupils and pupils of SSHIs are shown in Tables 1, 2.

**Table 1:** **Medical and hearing variables for students in regular classes of secondary mainstream schools, integrative classes of secondary mainstream schools and students in secondary special schools for hearing impaired (SSHI)**.

	**Mainstream regular (*n* = 38)**	**Mainstream integrative (*n* = 19)**	**SSHI (*n* = 83)**
Causes of deafness, numbers (percent)			
Unknown, n (%)	27 (71%)	16 (84%)	52 (63%)
Meningitis/Rubella, n (%)	3 (8%)	1 (5%)	7 (8%)
Connexin 26, n (%)	1 (3%)	0 (0%)	3 (4%)
Mondini Dysplasia, n (%)	1 (3%)	0 (0%)	1 (1%)
Other Infections, n (%)	1 (3%)	0 (0%)	4 (5%)
Other Illnesses, n (%)	5 (13%)	2 (11%)	16 (20%)
Indications for additional handicaps n (%)^5^	8 (21%)	3 (16%)	24 (29%)
Age at first fitting of hearing aids in months, mean ± SD	20.95 ± 19.72	18.67 ± 10.77	20.39 ± 14.60
Benefit of hearing aids (minimal perception of acoustic stimuli with hearing aids) before CI^6^, mean ± SD	2.84 ± 1.26	2.79 ± 1.40	2.84 ± 1.27
Age at implantation of 1th CI in months, mean ± SD	50.67 ± 45.64	44.94 ± 40.00	60.19 ± 47.15
Late implantation (>60 months), n (%)	10 (26%)	3 (16%)	25 (30%)
Bilateral implantation, n (%)	19 (50%)	11 (58%)	45 (54%)
Age at implantation of 2nd CI in months, mean ± SD^**^	99.20 ± 44.31	138.27 ± 40.02	127.28 ± 32.17
Monosyllables 65 dB^7^ in %, mean ± SD	71% ± 18%	72% ± 15%	73% ± 26%
Understanding of sentences in noise^8^, n (%) ^***^	29 (76%)	14 (73%)	45 (54%)

** p < 0.01,

*** p < 0.001.

**Table 2:** **School variables, family variables and other demographic data of students in regular classes of secondary mainstream schools, integrative classes of secondary mainstream schools, and students in secondary special schools for hearing impaired (SSHI)**.

	**Mainstream regular (*n* = 38)**	**Mainstream integrative (*n* = 19)**	**Special HI (*n* = 83)**
**SCHOOL VARIABLES**			
**Sign language**			
Education, n (%)^***^ primarily	4 (11%)	1 (5%)	36 (43%)
Use, n (%)^***^	15 (39%)	10 (52%)	58 (70%)
Competence^a^, mean ± SD^***^	1.10 ± 1.39	3.52 ± 1.43	2.32 ± 1.33
Preference, n (%)	0 (0%)	1 (5%)	1 (1%)
**Additional teacher, n (%)**	5 (13%)	4 (21%)	12 (14%)
Residential school, n (%)^***^	2 (5%)	5 (26%)	33 (40%)
**FAMILY VARIABLES**			
**Social background**			
Mothers Skill level^b^, mean ± SD	1.81 ± 0.78	1.71 ± 0.99	1.63 ± 0.70
Fathers Skill level^b^, mean ± SD^*^	2.17 ± 0.45	2.26 ± 0.65	1.93 ± 0.51
Mothers Education^c^, mean ± SD	2.22 ± 0.92	2.05 ± 0.97	2.28 ± 1.34
Fathers Education^c^, mean ± SD	2.33 ± 1.16	2.63 ± 1.34	2.35 ± 1.25
Only Child, n (%)	7 (18%)	7 (37%)	18 (21%)
Single Parents, n (%)^*^	2 (5%)	4 (21%)	16 (19%)
**REGION IN GERMANY**			
Munich, n (%)	8 (21%)	1 (5%)	14 (16%)
Hannover, n (%)	14 (37%)	11 (58%)	18 (21%)
Mainz, n (%)	7 (18%)	2 (11%)	35 (42%)
Freiburg, n (%)	9 (24%)	5 (26%)	16 (19%)

* p < 0.05,

*** p < 0.001.

a Rated by the parents on a scale from 0 to 4. The higher the number the higher the competence level.

b Rated on a 4-point scale according to the “**I**nternational **S**tandard **C**lassification of **O**ccupation” (ISCO, International Labor Office, 1990). The higher the number the higher the parents' ISCO-Level.

c Rated by a 5-point scale according to the “**I**nternational **S**tandard **C**lassification of **Ed**ucation ((ISCED)” The higher the number the higher the parents' ISCED-Level. http://www.uis.unesco.org/Education/Pages/international-standard-classification-of-education.aspx (assessed 4.11.2015).

Regrettably only few teachers participated in the study.

The majority of students, who went to a secondary SSHI, also had been schooled in an SSHI primary (*n* = 67; 81%). The majority of the students who chose a secondary mainstream school (with- or without integration) had also been schooled in a primary mainstream school (*n* = 34; 60%).

### Instruments

Mental health problems were assessed with the “Strengths and Difficulties Questionnaire” (SDQ, Youth in mind, 2014) (Goodman, 1997). The SDQ evaluates emotional, behavioral and social problems of children and adolescents aged about 3–17 years. It can also be used as a screening measure for mental health disorders, which was not the case in the present study. Its good psychometric properties have been confirmed by many studies worldwide (Goodman et al., 2000; Muris et al., 2003; Becker et al., 2004a; Du et al., 2008). The brief 25 item rating scale addresses emotional symptoms (ES), inattention-hyperactivity (HA), conduct problems (CP), peer-problems (PP) and pro-social behavior (PBS), (social strengths, e.g., altruism). The scores of ES, HA, CP and PP are summarized in the “Total Difficulty Score” (TDS). The “impact supplement” in the extended version evaluates the impact of mental health problems on the well-being of young people, their everyday life and their functioning in family, at school, with friends and with peers. SDQ versions are available for parents, teachers and as self ratings for children from 11 years of age and older. There are three response categories: 0 = not true, 1 = somewhat true, and 2 = certainly true. Higher values mean more problems. The SDQ has been translated and validated for the German language (Becker et al., 2004b,a).

### Procedures

The participants were recruited on the occasion of the annual appointment in the clinics. Both, adolescents and their parents were asked to participate. All participating adolescents and their parents were surveyed individually. Additional audiological tests like speech perception tests in quiet and noise were performed. Medical data were obtained from clinic files. Other demographic data were collected by parental surveys. The patients completed the SDQ questionnaire under surveillance by a clinic member. In 16 cases support was needed, whereby the SDQ questions were additionally presented in an adapted format, with standard sentences—following a written guideline, shortened and with paraphrases, presented both orally and written. This support did not replace the original SDQ questionnaire. The use of a sign language interpreter was not required. The parents filled in the questionnaires (SDQ, demographic data) at the same time, however separately. In the case of their agreement, the teachers received the SDQ from the parents and sent it back to the investigators via mail. Teacher ratings were available for 55 adolescents with CI. Further information about the procedures can be found in Huber et al. (2015).

### Statistics

In order to analyze whether mental health problems of adolescents with CI differed between school types (Hypothesis a, see 3.1. for the results), we used multivariate ANOVAs with independent variable school type (special/regular) and dependent variables EP, CP, HA, and PP. Subsequent univariate analyses were performed to identify in which area the problems manifested. TDS, PBS, and SDQ impact were each compared between school types using One-way ANOVAs. All analyses were performed separately on self-, parent-, and teacher-ratings.

In order to analyze, which hearing variables (see Table 1), school variables (sign language, additional teacher, residential school, see Table 2) and family variables (social background, only child, single parent, see Table 2) differed significantly between school types (Hypotheses b-d, see section “Differences in Possible Explanatory Variables Between School Types” for the results) and were therefore possibly explanatory for the effect of school type. Comparisons between school types were conducted using independent samples *t*-tests, Mann-Whitney U-tests, Chi-Square tests or multivariate ANOVAs depending on the scaling and dimensionality of the variables.

In order to analyze, which of the variables differing between school types were explanatory for the effect of school type (Hypothesis e), mediation analyses were carried out. Therefore, we first identified, which variables had also a significant impact on mental health problems (see section “Relation of Possible Explanatory Variables to Mental Health Problems” for the results). Analyses were performed using multivariate ANOVAs, *t*-tests or Pearson-correlations depending on the scaling of the independent variable. Second, possible explanatory variables with a significant impact on mental health problems were entered as covariates in the comparison of school types, to see whether they were able to explain the effect of school type (see section “Effect of School Type after Controlling for Explanatory Variables” for the results).

### Ethical approval

The present study was approved by the ethics committees in Salzburg (Ethikkommission für das Bundesland Salzburg), Munich (Ethikkommission der LMU München), Mainz (Ethikkommission der Landesärztekammer Rheinland-Pfalz), Freiburg (Ethikkommission der Albert-Ludwigs-Universität Freiburg), and Hannover (Ethikkommission der Medizinischen Hochschule Hannover). According to the Declaration of Helsinki the study was carried out with written informed consent from all enrolled subjects.

## Results

### Differences in mental health problems between school types

**Figure 1:**
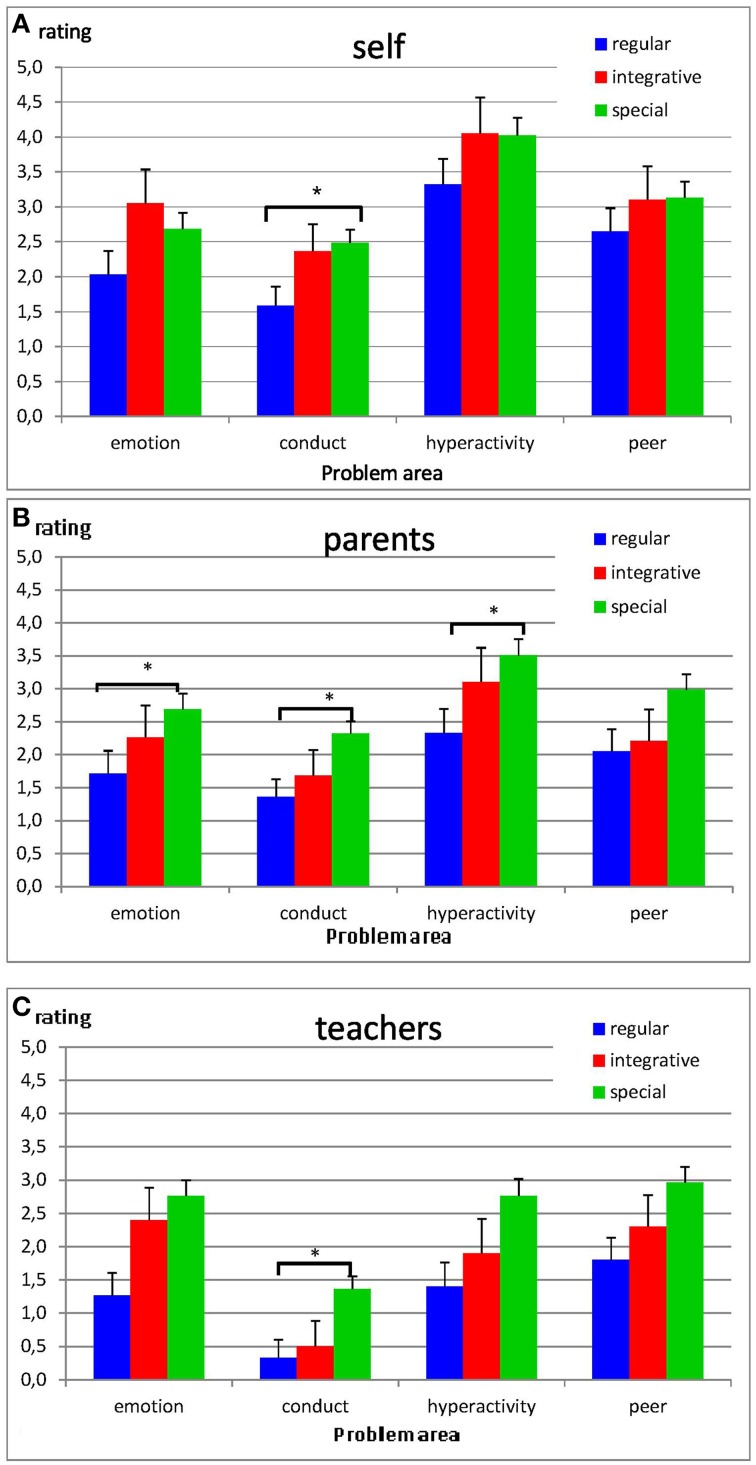
**SDQ self- (A), parent- (B), and teacher-ratings (C) for emotional problems, conduct problems, hyperactivity, and peer problems (means ± SE)**. Higher scores indicate more problems. Significantly different from regular schools: ^*^p < 0.05.

TDS differed significantly between school types in parents [*F*_(136)_ = 7.93, *p* < 0.001], self [*F*_(126)_ = 4.36, *p* < 0.05] and teacher ratings [*F*_(52)_ = 3.28, *p* < 0.05]. *Post-hoc* Tukey tests revealed significant differences between students in regular classes of secondary mainstream schools and students in SSHIs (all *p*_*post-hoc*_ < 0.05), but no differences between these school types and integrative classes of secondary mainstream schools. Students in secondary SSHIs had significantly more problems than students in regular classes of secondary mainstream schools (compare Table 3 and Figure 1). Students in integrative classes of secondary mainstream schools had slightly more problems than students in regular classes of mainstream schools, but less problems than students in SSHIs. SDQ impact did not differ between school types (all *F* < 2.85, all *p* > 0.06).

**Table 3:** **Means ± SE of SDQ self-, parents-, and teacher ratings**.

	**Total difficulties**	**Emotional symptoms**	**Conduct problems**	**Hyperactivity inattention**	**Peer problems**	**SDQ impact**	**Prosocial behavior**
**SELF**							
Regular	9.59±0.64	2.03±0.32	1.59±0.25	3.32±0.31	2.65±0.30	1.13±0.23	8.29±0.23
Integrative	12.58±1.81	3.05±0.43	2.37±0.34	4.05±0.42	3.11±0.40	1.25±0.37	7.68±0.43
SSHI	12.33±0.47^*^	2.68±0.21	2.49±0.17^*^	4.03±0.21	3.13±0.20	1.22±0.19	7.49±0.17^*^
**PARENTS**							
Regular	7.46±0.78	1.72±0.34	1.36±0.27	2.33±0.37	2.05±0.34	0.47±0.15	8.23±0.34
Integrative	9.26±1.40	2.26±0.49	1.68±0.39	3.11±0.52	2.21±0.48	1.16±0.42	8.00±0.38
SSHI	11.51±0.59^**^	2.69±0.24^*^	2.32±0.19^*^	3.51±0.25^*^	2.99±0.23	1.20±0.19	7.84±0.19
**TEACHERS**							
Regular	4.80±1.02	1.27±0.63	0.33±0.36	1.40±0.67	1.80±0.70	0.20±0.11	7.87±0.58
Integrative	7.10±1.86	2.40±0.77	0.50±0.45	1.90±0.83	2.30±0.85	0.67±0.29	7.10±0.77
SSHI	9.87±1.35^*^	2.77±0.46	1.37±0.26^*^	2.77±0.48	2.97±0.49	1.03±0.26	7.23±0.40

Higher scores indicate moreproblems, exception prosocial behavior (lower scores indicate more problems). Significantly different from regular classes of mainstream schools:

* p < 0.05,

*** p < 0.001. Regular, Regular classes of mainstream schools; Integrative, Integrative classes of mainstream schools; SSHI, Special schools for hearing impaired.

In the multivariate analysis a significant main effect of secondary school type was observed for parent ratings (parents: [*F*_(8, 268)_ = 1.99, *p* < 0.05]), but not for self and teacher ratings (both *F* < 1.39, both *p* > 0.20). Post-hoc Tukey tests were performed to evaluate to which problem areas the differences were attributable. Parent ratings differed significantly between regular classes of secondary mainstream schools and SSHIs in emotional, conduct and hyperactivity problems (all *p*_*post-hoc*_ < 0.05). Self and teacher ratings differed significantly between regular classes of secondary mainstream schools and SSHIs in conduct problems (both *p*_*post-hoc*_ < 0.05), but not in emotional, hyperactivity or peer problems. Again no differences were observed between either school type and integrative classes of mainstream schools.

Prosocial behavior (PBS) did not differ between school types according to parent and teacher ratings (both *F* < 0.60, both *p* > 0.55). However, according to students' self ratings, PBS differed significantly between school types [*F*_(126)_ = 3.38, *p* < 0.05]. A *post-hoc* Tukey test revealed that proscocial behavior was rated significantly lower by students in SSHIs compared to students in regular classes of mainstream schools (p_*post-hoc*_ < 0.05) with no differences between these school types and integrative classes of mainstream schools (compare Table 3). Consequently, students visiting integrative classes of mainstream schools were excluded from all subsequent analyses.

### Differences in possible explanatory variables between school types

Tables 1, 2 list the values of possible explanatory variables by school types. Significant differences were identified in hearing variables (understanding of speech in noise, age at implantation of 2nd CI in the case of bilateral implantation and functional gain), but also in sign language (education, use and competence), residential school experience and social background (skill level and education of the parents) and single parenting.

### Relation of possible explanatory variables to mental health problems

#### Hearing variables

As demonstrated previously (Huber et al., 2015), understanding of speech in noise was significantly related to TDS parent ratings. However, the problem area most affected were peer problems, not conduct problems. Additionally, age at implantation of the 2nd CI was significantly related to self ratings of hyperactivity problems (*r* = 0.29, *p* < 0.05), but not to any other problem area. As bilateral implantation was significantly related to the ability to hear and understand speech in noise (*X*^2^ = 10.91, *p* < 0.001), but only about half of the adolescents were implanted binaurally, the mediating influence of the age at the implantation of the 2nd CI was not considered further.

#### School variables

##### Sign language

Since the majority of students, educated mainly in sign language, visited SSHIs, the impact of sign language was only analyzed among students who visited such schools. No significant differences in TDS, PBS, SDQ impact or any problem area were observed between students who had been educated in sign language and students who had only been educated in oral language (all *t* < 1.77, all *p* > 0.08), with the exception of teacher ratings of conduct problems [*t*_(28)_ = 2.48, *p* < 0.05] with more problems for sign language educated students. Furthermore, no significant correlation between sign language competence and TDS or any problem area was observed.

##### Residential school experience

Since the majority of students with residential school experience visited SSHIs, the impact of residential school experience was only analyzed among students who visited secondary SSHIs. Self-, parent- or teacher-ratings of mental health problems did not differ significantly between SSHI students in residential schools and SSHI students who lived at home (all |t| < 1.92, all *p* > 0.06). Furthermore, the total duration of residential school experience was not correlated to either self-, parents or teacher ratings of mental health problems (all |r| < 0.22, all *p* > 0.19).

#### Family variables

##### Social background

Students self-ratings of conduct problems were significantly negatively correlated with the fathers' skill level (*r* = −0.24, *p* < 0.05). Parents ratings of conduct problems were significantly negatively correlated with the mother's skill level (*r* = −0.24, *p* < 0.05). The higher the parents skill level, the less severe conduct problems were reported. No correlation was observed between conduct problems and parents education (all |r| < 0.12, all *p* > 0.27). No other area of mental health problems was correlated to social background variables in self-, parent- or teacher ratings (all |r| < 0.12, all *p* > 0.25).

##### Only children

No significant differences were observed in either self-, parents- or teacher-ratings of mental health problems between only children and children with siblings (all |t| < 1.62, all *p* > 0.10).

##### Single parenting

Since the majority of students from single parent families went to SSHIs, the impact of single parenting was only analyzed for students in this type of school. No significant effects of single parenting on TDS were observed in self- and parent-ratings (both |t| < 1.58, both *p* > 0.11), but TDS teacher ratings were significantly higher for students from single parent families (*t* = −2.07, *p* < 0.05). A significant main effect of single parenting on mental health problems was observed in the multivariate analyses of parent ratings [*F*_(4.68)_ = 2.68, *p* < 0.05] but not for self and teacher ratings [*F*_(4.71)_ = 2.05, *p* = 0.08; *F*_(4.35)_ = 1.35, *p* = 0.28]. Univariate analyses revealed that the effects on parent ratings were attributable to conduct problems [*F*_(1.79)_ = 6.20, *p* < 0.05], with no effect of single parenting on the other problem areas (all *F* < 3.28, all *p* > 0.08). No effect of single parenting was observed on SDQ impact in either rater (all *t* < 1.59, all *p* > 0.11). No effect of single parenting was observed on parent and teacher ratings of PBS (both *t* < 0.73, both *p* > 0.47). However, self ratings of PBS were significantly lower in students from single parent families [*t*_(74)_ = 2.09, *p* < 0.05].

### Effect of school type after controlling for explanatory variables

In summary, the following variables were identified as potential explanatory variables for the effect of school type (regular class in secondary mainstream school or secondary SSHI): (a) hearing (ability to hear and understand speech in noise), (b) social background (fathers skill level), (c) single parenting.

These variables were now each entered as covariates into the multivariate analysis of school type, in order to evaluate whether differences in mental health problems between school types were diminished, when the influence of the covariate was taken into account (Table 4).

**Table 4:** **F-values for differences between school types (mainstream school, special school of persons with hearing loss) without covariates (first column) and after controlling for (i) ability to hear and understand in noise (ii) social background (fathers skill level), (iii) single parenting as well as (iv) all three variables simultaneously**.

**Covariate**	**None**	**Hearing**	**Social background**	**Single parenting**	**all**
**SELF RATINGS**					
TDS	11.13^***^	6.84^**^	7.61^**^	10.05^**^	4.44^*^
Multivariate analysis	3.27^*^	2.37	2.01	2.89^*^	1.25
EP	3.30	3.90	4.34^*^	2.20	2.65
CP	10.60^**^	6.72^*^	4.67^*^	8.87^**^	2.90
HA	4.13^*^	2.56	2.30	5.34^*^	1.99
PP	2.12	0.36	1.97	1.98	0.64
Impact	0.07	0.02	0.26	0.02	0.02
PBS	7.45^**^	6.61^*^	4.68^*^	5.77^*^	3.70
**PARENT RATINGS**					
TDS	16.13^***^	12.25^***^	12.04^***^	13.82^***^	6.39^*^
Multivariate analysis	4.02^**^	3.20^*^	3.00^*^	3.45^*^	1.70
EP	5.78^*^	3.56	5.01^*^	4.29^*^	1.57
CP	9.96^**^	9.44^**^	7.63^**^	7.28^**^	5.43^*^
HA	7.09^**^	4.11^*^	4.65^*^	7.10^**^	2.42
PP	5.23^*^	4.33^*^	3.47	5.27^*^	1.85
Impact	5.78^*^	3.95^*^	5.84^*^	4.36^*^	1.59
PBS	1.17	3.41	1.95	1.07	2.49
**TEACHER RATINGS**					
TDS	6.14^*^	3.45	5.29^*^	4.94^*^	1.00
Multivariate analysis	1.59	0.88	1.38	1.29	0.28
EP	3.84	2.35	3.97	2.84	0.79
CP	4.76^*^	2.18	4.12^*^	3.83	0.55
HA	2.80	1.16	2.01	2.01	0.23
PP	1.83	1.70	2.02	1.53	0.48
Impact	4.87^*^	2.71	3.69	3.90	0.84
PBS	0.83	0.54	1.25	0.81	0.32

* p < 0.05,

** p < 0.01,

*** p < 0.001.

These ANCOVAs revealed that each explanatory variable, entered as a covariate on its own, slightly reduced the differences between school types. However, none of these variables was able to completely explain all differences between school types, since in each ANCOVA some differences between the school types remained. Differences between school types persisted, even after the variables were entered as covariates. However, most differences disappeared when all variables were simultaneously entered as covariates in the comparison of school types. However, even then, some differences still remained, e.g., self- and parent rated TDS; compare Table 4 last column.

## Discussion

In a multicenter study on hearing impaired adolescents with CI we compared the extent of mental problems between pupils of regular classes of secondary mainstream schools, pupils of integrative classes of secondary mainstream schools and pupils of secondary special schools for hearing impaired (SSHIs).

Students of SSHIs showed significantly more conduct problems (CP) and a significantly higher TDS compared to pupils of regular classes of mainstream schools (self-, parent-, and teacher rating, each). Furthermore, they showed significantly more emotional symptoms (ES parents), a higher level of hyperactivity (HA, parents) and more problems with prosocial behavior (PBS, self). Students in integrative classes in mainstream schools showed more problems than pupils of regular classes and fewer problems than pupils of SSHIs. However, these latter differences were not significant.

These outcomes confirm the first hypothesis of the present study. Students with CI in SSHIs show more mental health problems than students with CI in mainstream schools. Additionally they corroborate the results of earlier studies on young hearing impaired persons with (Huber and Kipman, 2011) and without CI (van Eldik, 2005; Mejstad et al., 2009; Remine and Brown, 2010; Theunissen et al., 2014a).

All three informants, i.e., self (adolescents), parents and teachers agreed that pupils of SSHI have significantly more conduct problems and a significantly higher TDS compared to mainstream pupils. Differences in emotional symptoms, hyperactivity (parents) and prosocial behavior (self) were only found in ratings of one informant. Becker et al. (2004b) demonstrated that the predictive quality of the SDQ for mental illness or mental health disorders^9^ is only satisfying in the case of an agreement between all three informants. In the case of disagreement the predictive quality of the SDQ was only moderate, especially if only the rating of a single informant was significant (Becker et al., 2004b). Therefore, the outcomes about the increased rates of conduct problems and a higher TDS of students attending SSHIs are more informative and have a higher scientific value than the results regarding emotional symptoms, hyperactivity and prosocial behavior problems (disagreement).

The increased rate of conduct problems in students at SSHIs indicates an increased risk for conduct disorders and further behavioral and/or learning disorders. According to the American Academy of Child and Adolescent Psychiatry (AACAP) “*many children with a conduct disorder may have coexisting conditions such as mood disorders, anxiety, PTSD (Posttraumatic stress disorder) substance abuse, ADHD” and “learning problems. Without treatment, many youngsters with conduct disorder are unable to adapt to the demands of adulthood and continue to have problems with relationships and holding a job.”* As shown by studies on hearing impaired children without CI, conduct problems and other behavioral problems start in the first years of life (Barker et al., 2009) and continue during childhood (Stevenson et al., 2010). They are associated with difficulties in oral language (Barker et al., 2009; Stevenson et al., 2010), communication difficulties (Barker et al., 2009), and furthermore with attention problems (Barker et al., 2009).

We also examined the hypotheses, that SSHIs are mainly attended by problematic adolescents and that these problems may explain the relationship between school type and mental health problems of adolescents with CI.

First, it was only partly confirmed, that SSHI are mainly attended by somewhat more difficult cases. CI users with a late 2nd CI, CI users, who are distinctly restricted to understand speech in noise, CI users with a disadvantaged social background (skill level of the father) as well as CI users from single-parent families were overrepresented in SSHIs. However, adolescents with indications for additional handicaps as well as late implanted adolescents (1st CI) were equally distributed in both school types. Also speech perception outcomes in quiet did not differ between school types.

Second, it was also only partly confirmed that mental health problems of CI adolescents in SSHIs were explained by these disadvantages. Understanding of speech in noise, skill level of the father and single parenting were found to possess some amount of impact, but none of these variables explained comprehensively the differences in mental health problems between SSHIs and mainstream schools. Most likely a combination of all three variables may play a role for the differences between the two school types. Regarding the speech in noise outcomes, mean scores would have been more informative. However, due to the different audiological sentence tests the participating centers used to evaluate speech perception in noise, we were restricted to the dichotomic assessment of the audiologists (basing on the actual outcomes of the adolescents).

Third, already existing mental health problems could have caused parents to choose an SSHI environment for their child. In the present study more than 80% of students, who went to a secondary SSHI, also had been schooled in an SSHI primary. This could indicate, that the majority of adolescents had already shown mental health problems at a very young age. Because of the retrospective study design, it is however not possible to clearly answer this question. The few studies addressing mental health problems of children with CI before school age (Martin et al., 2011; Wiefferink et al., 2012) indicate that very young CI children are less socially competent than normal hearing children (Wiefferink et al., 2012) and that they show some peer problems (Martin et al., 2011). However, there is no indication for more problems in externalizing behaviors like hyperactivity or conduct problems (Wiefferink et al., 2012).

However, additional variables that have not been addressed in the present study, may also account at least in part for the increased rate of mental health problems of students of SSHI including lexical and syntactical knowledge as well as communication skills (compare e.g., Theunissen et al., 2014b).

Furthermore, the feeling to be strange or not “normal” and loneliness may play a role in pupils of SSHIs. In Germany and Austria pupils of SSHIs come from a large geographical area. Because these schools are usually not located in the neighborhood of the children/adolescents, it is difficult for commuters between school and home to meet classmates after school or at the weekend. In normal hearing children loneliness in childhood predicts problems with social adjustment, depression, aggression, and suicidal thoughts in adolescence (Schinka et al., 2013).

## Conclusion

Young CI users who attend special schools for hearing impaired have more mental health problems than adolescents with CI who attend regular classes in mainstream schools. Students at special schools for hearing impaired have additional problems. They are more restricted to understand speech in noise, the age at implantation of the 2nd CI is higher, the socioeconomic status is lower, and the adolescents are more likely to come from single-parent families. The variables speech understanding in noise, fathers skill level and single parenting were found to be partial mediators of the differences in mental health problems between the two school types. However, none of these variables could explain comprehensively, why students of special schools for hearing impaired have more mental health problems than mainstream pupils.

## Author contributions

MH was the leading investigator. She developed the proposal for the multi-center study, organized the funding and converted the centers to the cooperation. She played a leading role in the composing of this paper. As first author she is primarily accountable for all aspects of the work. BP was responsible for the statistical analysis and the results section. She also gave input on the structure of the introduction and discussion section and edited the paper for grammar and style. AG contributed to the acquisition of data, to the revision of the work, to ensuring that the questions of the work are appropriately investigated and to the approval of the version to be published. SK gave substantial contributions to the acquisition, analysis and interpretation of data for the work, to revising it critically for important intellectual content, to the final approval of the version to be published and her agreement to be accountable for all aspects of the work in ensuring that questions related to the accuracy or integrity of any part of the work are appropriately investigated and resolved. AN gave substantial contributions to the conception of the work, to the acquisition of data for the work, regarding critical revisions of the manuscript and the final approval of the version to be published. AI contributed to the acquisition of the patient data, the interpretation of data and she drafted parts of the paper. Finally she approved the versions to be published. She is accountable for all aspects of the work in ensuring, that questions related to the accuracy or integrity of any part of the work are appropriately investigated and resolved.

### Conflict of interest statement

Alexandros Giourgas, Angelika Illg, Andreas Nickisch and Silke Kunze are affiliated to kbo-Kinderzentrum München and Hannover Medical School, which have received research grants from Cochlear. The authors, themselves, did not receive person grants from Cochlear. Therefore they, and the other authors state that the research was conducted in the absence of any commercial or financial relationships that could be construed as a potential conflict of interest.

## Footnotes

^1^German schools fall within the competences of the federal states. This leads to differences in the organization of each federal state's particular school system. Basically, there is a distinction between Primary Education, Secondary Education I, and Secondary Education II. Primary Education mainly comprises elementary schools (Grundschule; grades 1–4). Secondary Education I comprises main schools (Hauptschule), middle schools (Realschule), comprehensive schools (Gesamtschule), and academic high schools (Gymnasium; grades 5–10), leading to the qualification for subject- related university (Fachhochschule). Secondary Education II comprises the stages 11–13 in academic high schools and qualifies for university.

^2^*Mental health* is defined by the WHO “as a state of well-being in which every individual realizes his or her own potential, can cope with the normal stresses of life, can work productively and fruitfully, and is able to make a contribution to her or his community” WHO (2014) (http://www.who.int/features/factfiles/mental_health/en/) (assessed 30.11.2015). *Mental health problems* are described on the focus of behavior as emotional, behavioral and social problems. Examples of other mental health approaches: *HRQOoL* (health related quality of life), with the focus on the (subjective) personal experience. *Mental health Disorders* have to fulfill nosological criteria as described in ICD 10 (World Health Organization, 2010). International Statistical Classification of Diseases—ICD 10. http://apps.who.int/classifications/icd10/browse/2010/en (assessed 19.2.2014). or DSM 5 (American Psychiatric Association, 2013).

^3^“Total Communication (TC) is a philosophy of educating children with hearing loss that incorporates all means of communication; formal signs, natural gestures, fingerspelling, body language, listening, lipreading, and speech” http://www.handsandvoices.org/comcon/articles/totalcom.htm (assessed 29.11.2015).

^4^Thirty-eight did not participate, as many girls as boys. Twenty-one families excused themselves because of time reason, 17 did not react to the invitation per mail.

^5^Information about the collection of these data can be found in Huber et al. (2015).

^6^According to the rating of the parents (4 point rating scale: 1, some profit; 4, no profit at all).

^7^Freiburger speech test (in quiet) (Hahlbrock, 1953).

^8^Information of the audiologists 0, is understanding; 1, is not understanding (basing on the actual outcome of the person with CI).

^9^**Mental health disorders** are fulfilling nosological criteria as described in ICD 10 (**I**nternational **S**tatistical **C**lassification of **D**iseases) or DSM 5 (**D**iagnostic and **S**tatistical **M**anual of Mental Disorders) American Psychiatric Association (2013) International Statistical Classification of Diseases—ICD 10.http://apps.who.int/classifications/icd10/browse/2010/en (Assessed September 19, 2014).
